# Porcine interferon lambda 3 (IFN-λ3) shows potent anti-PRRSV activity in primary porcine alveolar macrophages (PAMs)

**DOI:** 10.1186/s12917-020-02627-6

**Published:** 2020-10-28

**Authors:** Jun Zhao, Ling Zhu, Lei Xu, Jianbo Huang, Xiangang Sun, Zhiwen Xu

**Affiliations:** 1 College of Veterinary Medicine, Sichuan Agricultural University, Cheng Du, Sichuan Province China; 2 Key Laboratory of Animal Disease and Human Health of Sichuan Province, Sichuan Agricultural University, Cheng Du, Sichuan Province China

**Keywords:** IFN-λ3, Primary PAMs, PRRSV, Antiviral activity

## Abstract

**Background:**

Porcine reproductive and respiratory syndrome virus (PRRSV) is a serious viral disease of swine. At present, there are vaccines for the control of PRRSV infection, but the effect is not satisfactory. The recombination of attenuated vaccines causes significant difficulties with the prevention and control of PRRSV. Type III interferons (IFNs), also called IFN-λs, were newly identified and showed potent antiviral activity within the mucosal surface and immune organs.

**Results:**

Therefore, primary porcine alveolar macrophages (PAMs) were used for this investigation. To this end, we found that the replication of PRRSV in PAMs was significantly reduced after pre-treatment with IFN-λ3, and such inhibition was dose- and time-dependent. The plaque formation of PRRSV abrogated entirely, and virus yields were reduced by four orders of magnitude when the primary PAMs were treated with IFN-λ3 at 1000 ng/ml. In addition, IFN-λ3 in our study was able to induce the expression of interferon-stimulated genes 15 (ISG15), 2′-5′-oligoadenylate synthase 1 (OAS1), IFN-inducible transmembrane 3 (IFITM3), and myxoma resistance protein 1(Mx1) in primary PAMs.

**Conclusions:**

IFN-λ3 had antiviral activity against PRRSV and can stimulate the expression of pivotal interferon-stimulated genes (ISGs), i.e., ISG15, Mx1, OAS1, and IFITM3. So, IFN-λ3 may serve as a useful antiviral agent.

**Supplementary Information:**

The online version contains supplementary material available at 10.1186/s12917-020-02627-6.

## Background

Type I Interferons (IFN-α/β) and type III IFNs (IFN-λs), as the first line of defence in innate immunity, play a crucial role in the body’s resistance to exogenous pathogens [1]. Type III IFNs, also called IFN-λs, were first described in 2003 [2, 3], consist of IFN-λ1, IFN-λ2, IFN-λ3 and IFN-λ4 in humans [4, 5], IFN-λ2 and IFN-λ3 in mice [6, 7], IFN-λ1 and IFN-λ3 in swine [8–10]. Both IFN-α/β and IFN-λs bind to unique receptors and induce the previous signalling pathway and expression of the IFN-stimulated genes (ISGs) to mediate antiviral activity. Type I IFN interacts with a receptor formed by Interferon alpha/beta receptor 1 (IFNAR1) and Interferon alpha/beta receptor 2 (IFNAR2). Type III IFNs bind to the specific receptor chain IFN-λR1 and IL-10R2 [11]. Type I IFN receptor is ubiquitously expressed in various types of cells and organs; However, IFN-λR1 is widely expressed on epithelial cells, dendritic cells, human peripheral blood monocytes or macrophages [2], which means that IFN-λs may provide a focused antiviral response against mucosal and immune organ infections.

Interferon bind to its receptors on the cell surface and induce the production of a large number of ISGs, including ISG15, myxoma resistance protein (Mx) family, 2′-5′-oligoadenylate synthase (OAS) family and the IFN-inducible transmembrane (IFITM) family, through JAK-STAT signal transcription [12]. ISG15 is one of the most highly induced ISGs, ISG15 can inhibit viral translation, replication, or egress [13]. Mx1 is a broadly inhibitor and inhibits a wide range of viruses by blocking the endocytic traffic of incoming virus particles and the uncoating of ribonucleocapsids [14]. OAS1 can recognise the dsRNA produced by the virus in the infected cells and play an antiviral role by activating the ribonuclease L (RNase L) to degrade the diseased mRNA [15]. The IFN-inducible transmembrane (IFITM) family has a role in blocking virus entry [16]. IFITM3 has high potency against influenza A virus and severe acute respiratory syndrome (SARS) coronavirus [17].

PRRSV is a member of the family *Arteriviridae* in the order *Nidovirales*, which causes severe reproductive failure in sows and respiratory distress in piglets and growing pigs [18]. Also, PRRSV is an immunosuppressive virus that can infect the lymphatic system of the whole body and produce viraemia after infection [19]. PRRSV mainly infects and destroys porcine alveolar macrophages and leads to severe immunosuppression, which promotes the infection of *Mycoplasma pneumoniae*, *Streptococcus*, *A. pleuropneumoniae*, and other pathogens [20, 21]. The primary PAMs derived from piglet alveoli is an appropriate model for studying the interaction of PRRSV immune responses and host-pathogen in vitro*.* An in vivo antiviral test of type III interferons from pigs has not been reported. In this study, the antiviral activity of porcine IFN-λ3 against PRRSV in primary PAMs was evaluated, and the expressions of ISGs genes induced by IFN-λ3 was also investigated in primary PAMs.

## Results

### IFN-λ3 inhibits the replication of PRRSV in a dose-dependent manner in primary PAM cells

**Fig. 1:**
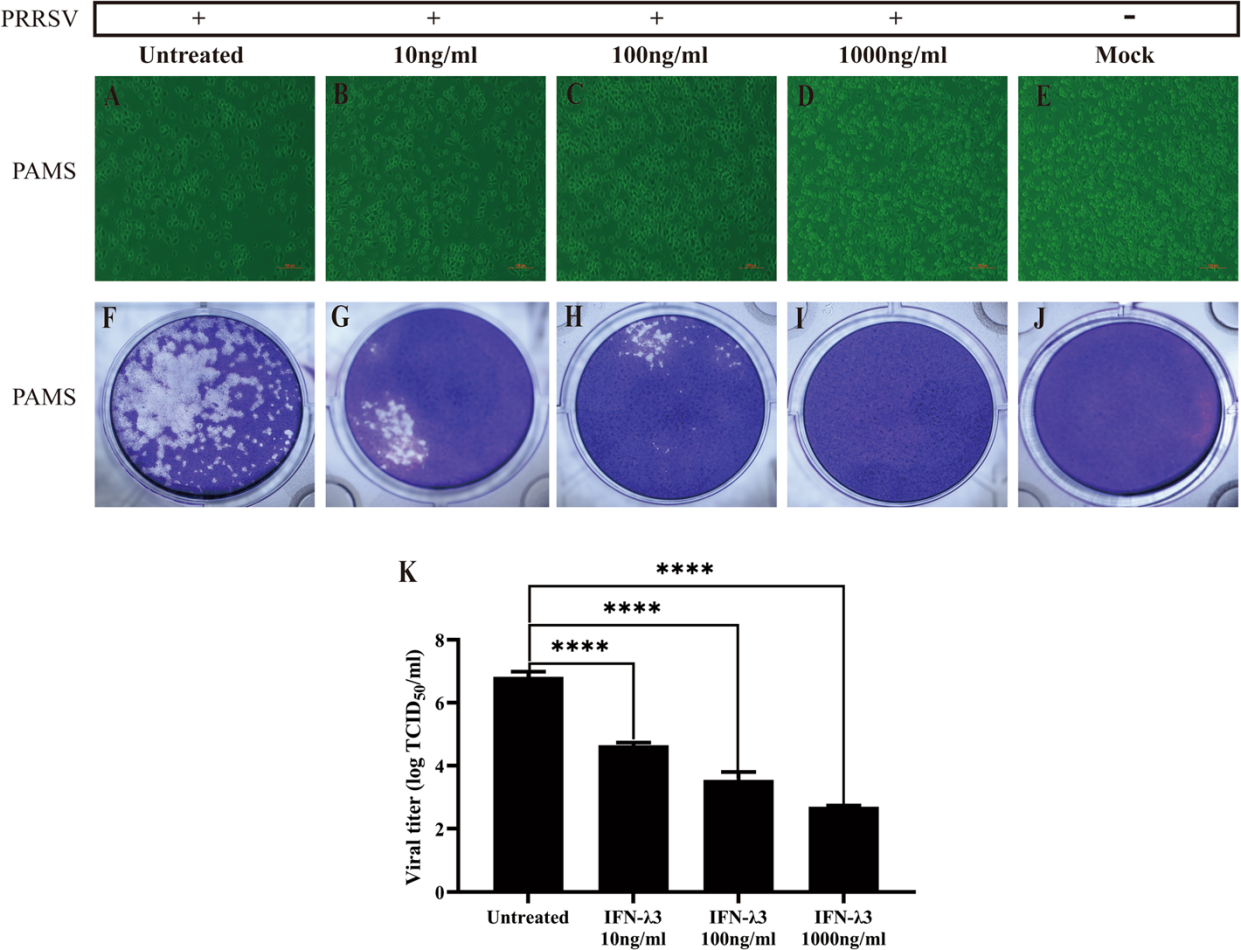
The CPE of primary PAMs treated with Porcine IFN-λ3 and infected with PRRSV. The primary PAMs were untreated or pre-treated with IFN-λ3 (10, 100, 1000 ng/ml). **b** The primary PAMs not treated. **b** The primary PAMs treated with 10 ng/ml IFN-λ3. **c** The primary PAMs treated with 100 ng/ml IFN-λ3. **d** The primary PAMs treated with 1000 ng/ml IFN-λ3. **e** Control primary PAMs. **k** The primary PAMs were treated or untreated with 100 ng/ml of IFN-λ3 for 12 h and then were infected with PRRSV NJ strain at 0.1 MOI. Infected cells were cultured for 12, 24, 36 or 48 h after infection. **f**, **g**, **h**, **i**, **j** corresponds to **a**, **b**, **c**, **d**, **e** with the same treatment. Magnifications, × 200

Previous study has confirmed that porcine IFN-λ3 possess the high specific activity against porcine epidemic diarrhoea virus (PEDV), classical swine fever virus (CSFV), hepatitis E virus (HEV) and so on [12, 22, 23]. In this study, we verified the antiviral effect of IFN-λ3 against PRRSV in vitro on PAMs. As shown in Fig. 1, treatment of primary PAMs with IFN-λ3 could reduce the multiplication of PRRSV. The degree of cytopathic effect (CPE) decreased with the increase in IFN-λ3 concentration (Fig. 1 A-E). The number and size of viral plaques also decreased with the increase in IFN-λ3 concentration (Fig. 1 F-J). The virus titre was significantly reduced with the increase of IFN-λ3 treatment dose (10, 100, 1000 ng/ml), and the maximum treatment dose could reduce the virus titre by four orders of magnitude compared with the control group (Fig. 1 K, the raw data are shown in supplementary Table S1). These results indicate that the IFN-λ3 could significantly inhibit the replication of PRRSV in a dose-dependent manner in primary PAMs.

### IFN-λ3 inhibits the replication of PRRSV in a time-dependent manner in primary PAM cells

**Fig. 2:**
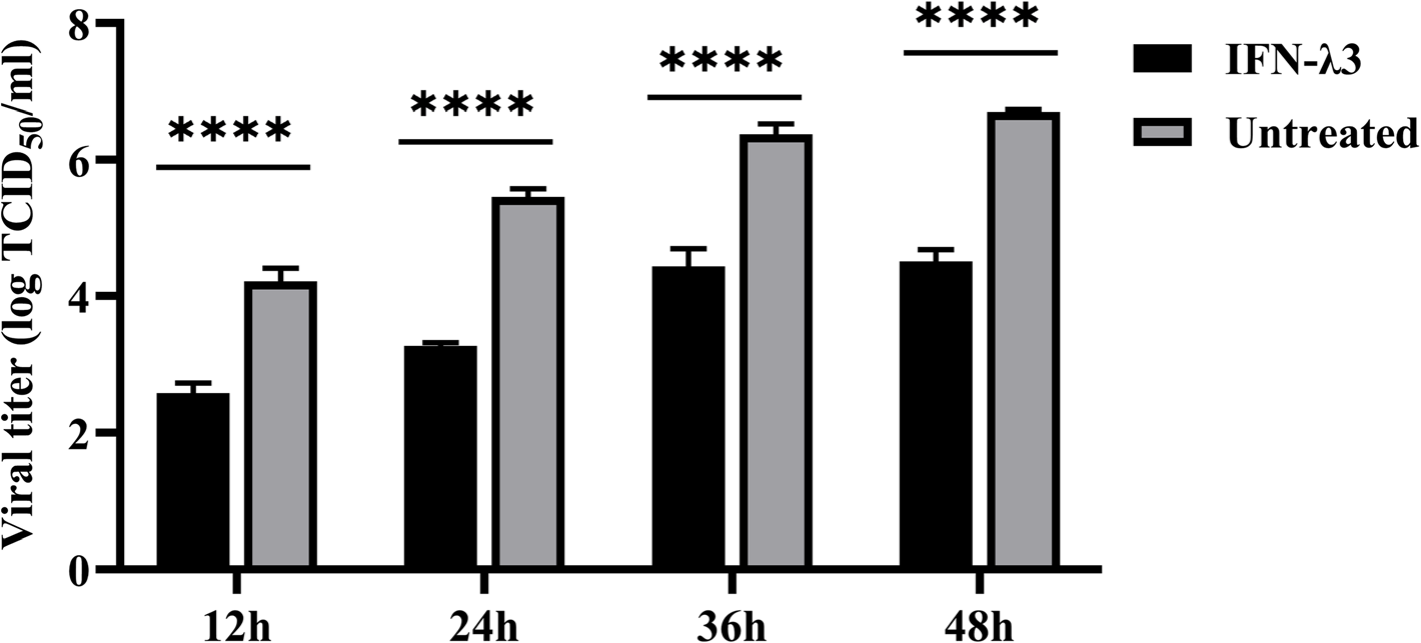
IFN-λ3 has antiviral activity against PRRSV and such inhibition is dose- and time-dependent in primary PAMs. The primary PAMs were stimulated with different doses of IFN-λ3 12 h, and then infected with PRRSV at 0.1 MOI. Infected cells were cultured for 48 h after infection. Note that the virus titre was titrated by TCID_50_. Data were presented as mean ± SEM (*N* = 3). *P < 0.05; **P < 0.01; ***P < 0.001; ****P < 0.0001 by Unpaired T-test

To investigate the time-dependent manner of the IFN-λ3 inhibits the replication of PRRSV, we used IFN-λ3 of 100 ng/ml concentration to treat PAMs cells and infected with the PRRSV. Cell cultures were collected at specific time and determine the virus titre. As shown in Fig. 2 (The raw data are shown in supplementary file 1, Table S2), the inhibition of IFN-λ3 on PRRSV decreased with time in primary PAMs, but the inhibition still existes. PRRSV proliferation slowed down within 36 h to 48 h in primary PAMs that were treated with IFN-λ3. The above results showed that IFN-λ3 could maintain a potent anti-PRRSV activity in the later stage and significantly inhibit the replication of PRRSV in a time-dependent manner in primary PAMs.

### IFN-λ3 inhibits PRRSV infection by activating ISGs in primary PAMs

**Fig. 3:**
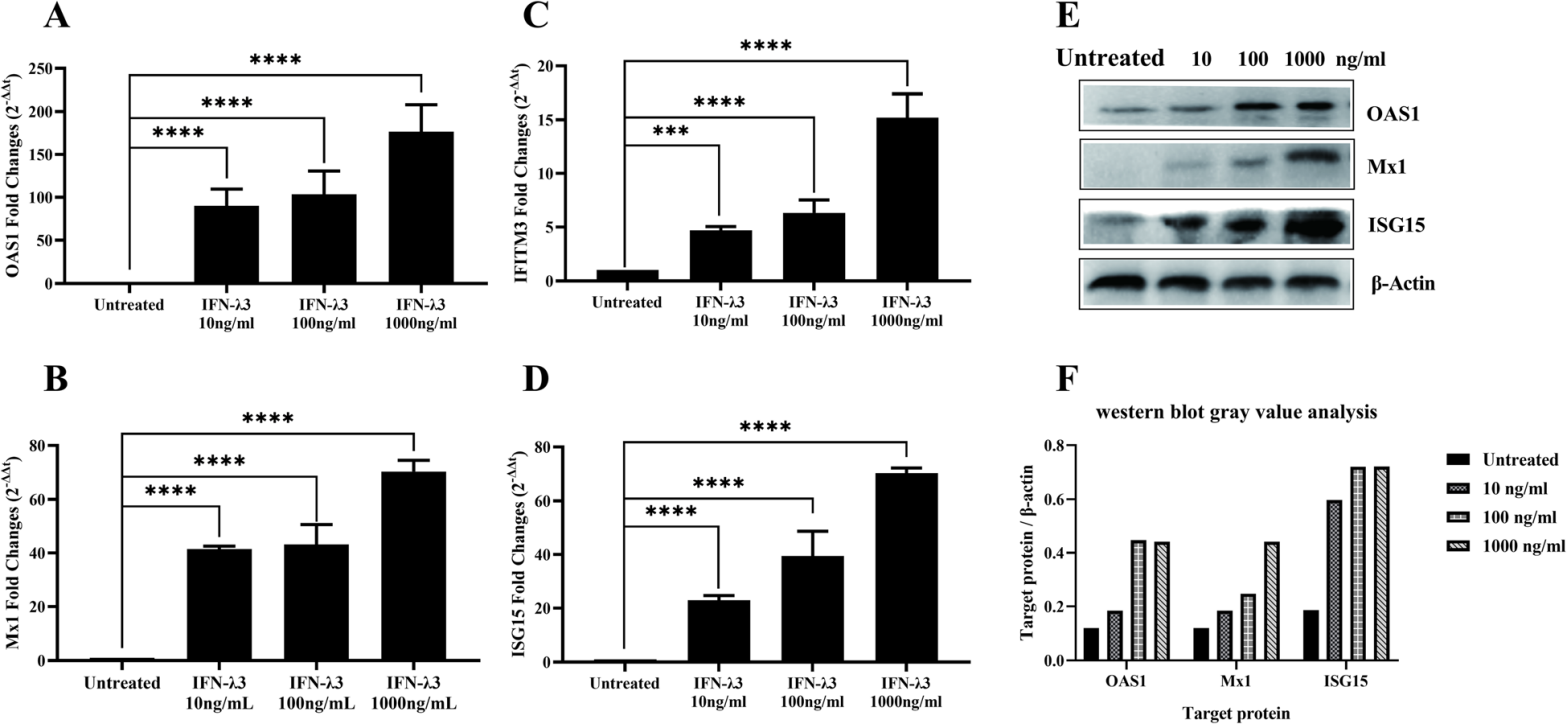
Expressions of ISGs induced by IFN-λ3 in primary PAMs. The primary PAMs were stimulated with different dose of IFN-λ3 12 h. qRT-PCR was performed to analyse the expression of OAS1(**a**), Mx1(**b**), IFITM3 (**c**), and ISG15 (**d**). The western blot was used to detect the proteins (**e**). The full-length blots are presented in Supplementary file 2 (a to d). The grey value of protein bands was measured by image J (**f**). Data were presented as mean ± SEM (*N* = 3). *P < 0.05; **P < 0.01; ***P < 0.001; ****P < 0.0001 by Unpaired T-test

ISG15, Mx1, OAS1 and IFITM3 have well-known antiviral properties and may affect PRRSV replication. Therefore, we accessed the quantification of ISGs induce by IFN-λ3. As seen in Fig. 3a to d, a dose-dependent induction of ISG15, Mx1, OAS1 and IFITM3 has been observed in primary PAMs treated with IFN-λ3. The expression of mRNA for ISG15, Mx1, OAS1, and IFITM3 was up-regulated by 70, 70, 160, and 15 times respectively at the concentration of 1000 ng/ml in primary PAMs. As shown in Fig. 3e and f (The full-length blots are presented in Supplementary file 2), a dose-dependent induction of the antiviral proteins ISG15, Mx1 and OAS1 has been observed in primary PAMs treated with IFN-λ3. The expression concentration of three antiviral proteins increased with the increasing IFN-λ3 concentration. Both ISG15 and Mx1 showed low expression in the untreated condition, and IFN-λ3 induced a large amount of expression. The expression of ISG15, Mx1 and OAS1 tended to be stable when the concentration of IFN-λ3 was higher than 100 ng/ml (Fig. 3f).

## Discussion

The results in our research confirm that the porcine IFN-λ3 shows potent anti-PRRSV activity in primary PAMs. PAMs are the first line of defence against pathogenic microbe infections in the lung. PRRSV replicates in monocytic lineage cell types, particularly in PAMs, and causes immunosuppression in swine [24]. Therefore, we selected the primary PAMs to carry out the IFN-λ3 in vitro anti-PRRSV study.

Alveolar macrophages are resident phagocytes of the alveolar space [24]. The expression of the IFN-λ receptor in alveolar macrophages has been confirmed and reported [25]. Macrophages express IL-10Rβ and IL-28Rα at both the mRNA and protein levels [26]. IFN-λ3 has the strongest antiviral function in IFN-λs [26]. In our study, the PRRSV proliferation reduced when primary PAMs were treated with IFN-λ3 (1000 ng/ml) (Fig. 1i). IFN-λ3 treatment could significantly reduce the virus titre of PRRSV proliferation on PAMs, and the virus titre of the 1000 ng/ml treatment group was four orders of magnitude lower than that of the control group (Fig. 2b). Consistent with these results, treatment with 10, 100 or 1000 ng/ml doses of recombinant human IFN-λ3 protected MARC-145 cells from PRRSV infection when compared to the untreated MARC-145 cells [27]. The study of two other kinds of viruses targeted porcine intestinal epithelial, PEDV and CSFV, confirming that IFN-λ3 inhibits their infection in vitro [22, 28]. All of these imply that porcine IFN-λ3 can inhibit the proliferation of porcine viruses such as CSFV, PEDV and PRRSV.

The antiviral activities of IFN-λ3 are due to ISG induction and IFN-λ3 can induce the expression of ISG. IFN-λ3 exerts its anti-HIV function by activating JAK-STAT pathway-mediated innate immunity in macrophages [29]. IFN-λ3 can bind to cell surface receptors and induce the high expression of interferon-stimulating genes of the MX, OAS and IFITM families [28, 30, 31]. The gene transcription profile induced by IFN-λ3, particularly the gene transcription profile induced by IFN-λ3 in primary PAMs has not been reported. In our study, we assessed whether the antiviral efficacy of IFN-λ3 was caused by the levels of ISG expression induced by IFN-λ3. Consistent with the expecting result, the expression of ISG15, OAS1, Mx1, and IFITM3 was provoked in primary PAM cells. The mRNA transcription and protein translation of the ISG15, OAS1 and Mx1 showed dose-dependence. However, the rangeability of mRNA and protein expression levels of ISG15, OAS1 and Mx1 were different. The expression levels of protein reached its peak when treated with 100 ng/ml IFN-λ3 while the expression of mRNA continuous increased (Fig. 3).

## Conclusion

In summary, our data demonstrated that IFN-λ3 could inhibit the replication of PRRSV in Primary PAMs, and such inhibition is dose- and time-dependent. Alveolar macrophages are one of the earliest immune defence cells in the lungs that contact pathogenic microorganisms. They are essential components of the innate and specific immunity of the host [32]. PAMs are an essential host cell for PRRSV natural infection. IFN-λ3 can stimulate the expression of pivotal ISGs, i.e. ISG15, Mx1, OAS1, and IFITM3. This study indicated that porcine IFN-λ3 might serve as a promising therapeutic agent against PRRSV and other viruses in swine in the future.

## Methods

### Virus and cells

African green monkey embryonic kidney epithelial MARC-145 cells were used to amplify PRRSV. MARC-145 cells were cultured with Dulbecco’s Modified Eagle’s medium (DMEM) (Gibco, USA) supplemented with antibiotics (100 units/ml of penicillin and 100 mg/ml of streptomycin) 10% heat inactivated foetal bovine serum (FBS) (Gibco, USA) at 37 °C under 5% CO_2_ atmosphere. PAMs were collected from lung lavages of 6-week-old Chenghua pigs (free of PRRSV, PCV2, PRV), as previously described, and cultured in RPMI-1640 (Gibco, USA) containing 10% FBS (Gibco, USA) at 37 °C [33]. PRRSV-NJ (NCBI GenBank accession No: MF818049.1) isolated from Sichuan province of China. The virus was propagated in MARC-145 cell with 2% FBS (Gibco, USA) added in the DMEM.

### Antiviral assay

To determine the anti-PRRSV activity of IFN-λ3 in the primary PAMs, the E. coli-derived IFN-λ3 was prepared in our laboratory. To explore the dose-dependent of IFN-λ3 antiviral, primary PAMs were untreated or pre-treated with IFN-λ3 (10, 100, 1000 ng/ml) for 12 h. Then, the cells were infected with PRRSV NJ strain at 0.1 MOI for 1–2 h, washed and replenished with fresh medium containing the indicated IFN-λ3. Infected cells were cultured for 48 h after infection. To explore the time-dependent effect of IFN-λ3 antiviral, primary PAMs were pre-treated with 100 ng/ml IFN-λ3 for 12 h. Then, the cells were infected with PRRSV NJ strain at 0.1 MOI for 1–2 h, washed and replenished with fresh medium containing the indicated IFN-λ3. Infected cells were cultured for 12, 24, 36, 48 h after infection. All of the cells were submitted to two freeze-thaw cycles and titrated by 50% tissue culture infective dose (TCID_50_) in Marc-145 cells. The cytopathic effect (CPE) units in culture plates were counted, and the viral titre analysis made use of the Reed-Muench Method. To examine the level of ISG expression in primary PAMs following IFN-λ3 stimulation, the cells were stimulated with the indicated concentrations (10, 100, 1000 ng/ml) of IFN-λ3 in 12-well plates for 12 h. Cells were then lysed, total RNA was extracted for subsequent qPCR analysis and total protein was extracted for western blot analysis. Every treatment group in this study had three duplicate samples (*N* = 3).

### Real-time quantitative PCR (qPCR)

Total RNA was extracted from the cellular supernatant or cell lysates using the EZ-10 Spin Column Total RNA Isolation Kit (Sangon Biotech (Shanghai) Co., Ltd., China) according to the manufacturer’s instructions and the RNA concentration was measured using a nucleic acid concentration analyzer (SCANDROP 200, Analytik Jena, Germany). Reverse transcription was performed using the Prime Script™ II 1st Strand cDNA Synthesis Kit (TAKARA), and qPCR was performed in a Light Cycler 96 (Roche, Switzerland) with TB Green® Premix Ex Taq™ II (Tli RNaseH Plus) (TAKARA). The thermal cycling conditions were 95 °C for 30 s, followed by 40 cycles of 95 °C for 5 s, and 60 °C for 30 s. All acquired data were obtained using Light Cycler 96 real-time PCR machines (Roche) and analysed with Light Cycler 96 software 1.5 based on the cycle threshold (ΔΔCT) method. Primers were designed using Oligo 6.0 software and are shown in Table 1.

**Table 1:** Primers used in this study

Genes	Primer names	Sequence (5′-3′)	Product size (bp)
Mx1	Mx1-F	CATCAACTTGGTGGTGGTC	200
	Mx1-R	CAATCATGTAGCCCTTCTTC	
β-actin	β-actin-F	ATCGTGCGGGACATCAAG	179
	β-actin-R	GGAAGGAGGGCTGGAA	
ISG15	ISG15-F	TGAGGGACTGCATGATGGC	197
	ISG15-R	CAGGATGCTCAGTGGGTCT	
IFITM3	IFITM3-F	GCTTCCCAGCCCTTCTTC	142
	IFITM3-R	TCTCGCTTCGGATGTTGAT	
OAS1	OAS1-F	TCCGAACGCAGGTCAAGG	136
	OAS1-R	AAGACGACGAGGTCAGCA	

### Western blot

Total protein was extracted from the cell lysates using the Western and IP Cell lysis Buffer (Sangon Biotech (Shanghai) Co., Ltd., China) according to the manufacturer’s instructions and protein concentration was determined using the BCA protein assay kit (Sangon Biotech (Shanghai) Co., Ltd., China). After gel electrophoresis, the proteins were transferred to nitrocellulose membranes (Bio-Rad, USA), and blocked in 5% skim milk at 4 °C overnight. After washing with PBST (0.5% Tween-20 in PBS), the membrane was incubated with primary antibodies for 2 h at 37 °C. After washing, the membrane was incubated with horseradish peroxidase (HRP)-conjugated IgG antibody (Abcam, No: ab170487) for 1 h at 37 °C. the protein bands were detected using SuperSignal™ West Pico PLUS Chemiluminescent Substrate (Thermo Scientific, USA) and chemiluminescence imaging system (BIO-RAD, ChemiDoc MP, California, USA). The primary antibodies of ISG15 (No: ab233071), OAS1(NO: ab86343), Mx1(No: ab95926) and β-actin (No: ab179467) was purchased from Abcam.

### Statistical analysis

Statistical analysis was performed and histogram were drawn using GraphPad Prism™ 8.0 (GraphPad Software, USA), Paired student *t*-test, and one-way ANOVA was used to test differences between different groups. *P* values< 0.05 were considered significant. The gray intensity of protein bolts was analyzed by Image J (National Institutes of Health, USA). The layouts and cropping of the pictures were completed by Adobe Illustrator CS6 (Adobe Systems Incorporated, California, USA).

## Supplementary Information

**Additional file 1: Table S1.** The Viral titer at 48 h after the PAMs stimulated with different dose of IFN-λ3. **Table S2.** The Viral titer at 12, 24, 36 or 48 h after the PAMs stimulated with IFN-λ3 (100 ng/ml).

**Additional file 2: Supplementary Figure** The antiviral proteins expression in PAMS treated with IFN-λ3 detected by Western blot. The expression of the OAS1, Mx1, ISG15 and β-actin proteins were detected by Western blot. d) Original blot images of OAS1, Mx1, ISG15 and β-actin in the Fig. 3e after treatment with IFN-λ3 (10, 100, 1000 ng/ml).

## Data Availability

All data generated during this study are included in supplementary file 1 (Table S1, S2). However, the raw data is available from the corresponding author upon reasonable request.

## Abbreviations

IFN-λ3: Interferon lambda 3
PAMs: Porcine alveolar macrophages
PRRSV: Porcine reproductive and respiratory syndrome virus
IFNs: Interferons
ISG15: Interferon-stimulated genes 15
OAS1: 2′-5′-Oligoadenylate synthase 1
IFITM3: IFN-inducible transmembrane 3
Mx1: Myxoma resistance protein 1
ISGs: Interferon-stimulated genes
IFN-α: Interferon alpha
IFN-β: Interferon beta
IFN-λs: Interferon lambdas
IFN-λ1: Interferon lambda 1
IFN-λ2: Interferon lambda 2
IFN-λ4: Interferon lambda 4
IFNAR1: Interferon alpha/beta receptor 1
IFNAR2: Interferon alpha/beta receptor 2
IFN-λR1: Interferon lambda receptor 1
IL-10R2: Interleukin 10 receptor 2
mRNA: Messenger RNA
SARS: Severe acute respiratory syndrome
PEDV: Porcine epidemic diarrhoea virus
CSFV: Classical swine fever virus
HEV: Hepatitis E virus
CPE: Cytopathic effect
IL-10Rβ: Interleukin 10 receptor beta
IL-28Rα: Interleukin 28 receptor alpha
HIV: Human immunodeficiency virus
MARC-145: African green monkey embryonic kidney epithelial cells
DMEM: Dulbecco’s modified eagle’s medium
FBS: Foetal bovine serum
PCV2: Porcine circovirus type 2
PRV: Pseudorabies virus
MOI: Multiplicity of infection
TCID50: 50% tissue culture infective dose
qPCR: Real-time polymerase chain reaction
PBST: 0.5% Tween-20 in PBS
HRP: Horseradish peroxidase
IgG: Immunoglobulin G
