# Supplementary material for: Porcine interferon lambda 3 (IFN-λ3) shows potent anti-PRRSV activity in primary porcine alveolar macrophages (PAMs)
Source: BMC Vet Res. 2020 Oct 28;16:408. doi: 10.1186/s12917-020-02627-6 (PMC7594293; doi:10.1186/s12917-020-02627-6)
Supplement: Supplementary file 1 — Additional file 1: Table S1. The Viral titer at 48 h after the PAMs stimulated with different dose of IFN-λ3. Table S2. The Viral titer at 12, 24, 36 or 48 h after the PAMs stimulated with IFN-λ3 (100 ng/ml). [file 12917_2020_2627_MOESM1_ESM.docx]

**Table S1.** The Viral titer at 48 h after the PAMs stimulated with different dose of IFN-λ3.

|  | Untreated | | | IFN-λ3  (10 ng/ml) | | | IFN-λ3 (100 ng/ml) | | | IFN-λ3 (1000 ng/ml) | | |
| --- | --- | --- | --- | --- | --- | --- | --- | --- | --- | --- | --- | --- |
|  | Ⅰ | Ⅱ | Ⅲ | Ⅰ | Ⅱ | Ⅲ | Ⅰ | Ⅱ | Ⅲ | Ⅰ | Ⅱ | Ⅲ |
| Viral titer (log TCID_50_/ml) | 7 | 6.8 | 6.66 | 4.73 | 4.66 | 4.55 | 3.66 | 3.25 | 3.73 | 2.66 | 2.71 | 2.73 |

**Table S2.** The Viral titer at 12, 24, 36 or 48h after the PAMs stimulated with IFN-λ3 (100 ng/ml).

| Time | log TCID_50_/ml | | | | | |
| --- | --- | --- | --- | --- | --- | --- |
|  | IFN-λ3 (100 ng/ml) | | | Untreated | | |
| 12h | 2.5 | 2.75 | 2.5 | 4 | 4.33 | 4.33 |
| 24h | 3.33 | 3.25 | 3.25 | 5.5 | 5.55 | 5.33 |
| 36h | 4.33 | 4.73 | 4.25 | 6.25 | 6.33 | 6.55 |
| 48h | 4.55 | 4.33 | 4.66 | 6.71 | 6.73 | 6.66 |
