# Supplementary material for: Porcine interferon lambda 3 (IFN-λ3) shows potent anti-PRRSV activity in primary porcine alveolar macrophages (PAMs)
Source: BMC Vet Res. 2020 Oct 28;16:408. doi: 10.1186/s12917-020-02627-6 (PMC7594293; doi:10.1186/s12917-020-02627-6)
Supplement: Supplementary file 2 — Additional file 2: Supplementary Figure The antiviral proteins expression in PAMS treated with IFN-λ3 detected by Western blot. The expression of the OAS1, Mx1, ISG15 and β-actin proteins were detected by Western blot. d) Original blot images of OAS1, Mx1, ISG15 and β-actin in the Fig. 3e after treatment with IFN-λ3 (10, 100, 1000 ng/ml). [file 12917_2020_2627_MOESM2_ESM.docx]

**Supplem. Figure Legends**

**The antiviral proteins expression in PAMS treated with IFN-λ3 detected by Western blot.**

The expression of the OAS1, Mx1, ISG15 and β-actin proteins were detected by Western blot.

1. d) Original blot images of OAS1, Mx1, ISG15 and β-actin in the Fig. 3E after treatment with IFN-λ3 (10, 100, 1000ng/ml).

**Supplem. Fig**


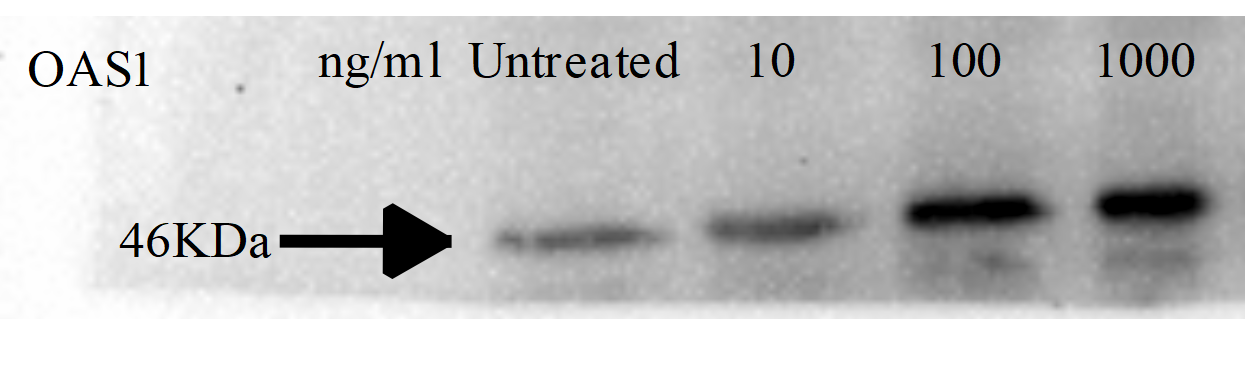


a


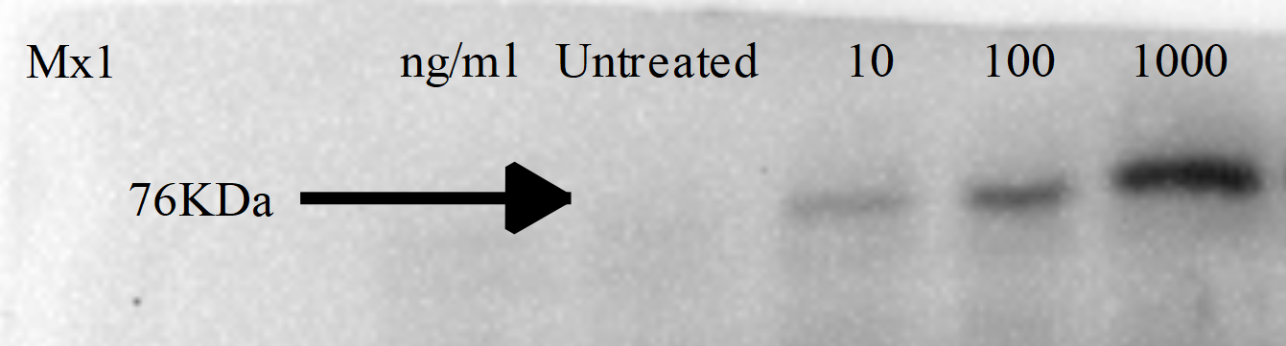


b


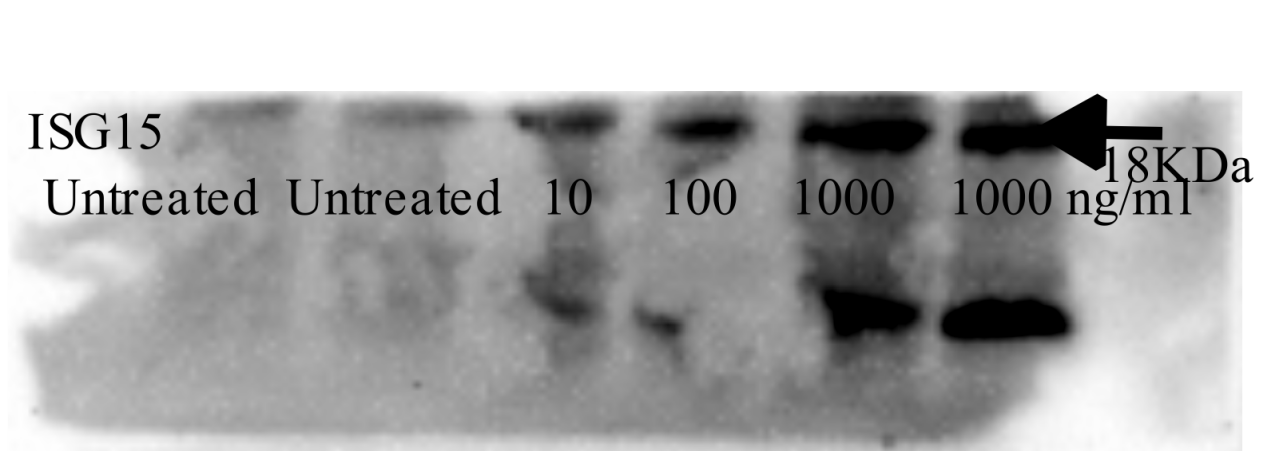


c


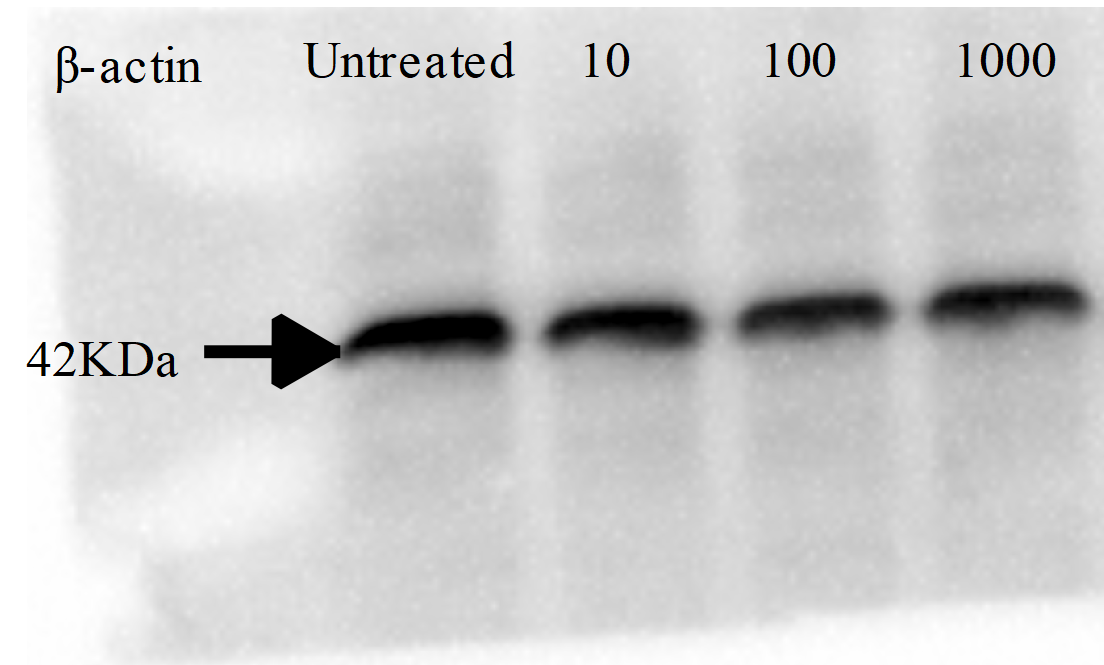


**ng/ml**

d
